# Dyskinesias and Treatment with Pramipexole in Patients with Parkinson's Disease

**DOI:** 10.1155/2012/473769

**Published:** 2012-02-09

**Authors:** John C. P. Piedad, Andrea E. Cavanna

**Affiliations:** ^1^Michael Trimble Neuropsychiatry Research Group, University of Birmingham and BSMHFT, Birmingham, UK; ^2^Department of Neuropsychiatry and Barberry National Centre for Mental Health, University of Birmingham and BSMHFT, 25 Vincent Drive, Birmingham B15 2FG, UK; ^3^Sobell Department of Motor Neuroscience and Movement Disorders, UCL, Institute of Neurology, London WC1N 3BG, UK

## Abstract

Dopamine agonists such as pramipexole (PPX) have first been proposed as adjunctive treatment to levodopa (L-DOPA) for patients with Parkinson's disease (PD) and then as a monotherapy alternative to alleviate dyskinesia. Treatment with PPX has overall been associated with improvement in parkinsonian symptoms. Although the majority of placebo-controlled studies demonstrated that dyskinesia was more prevalent in the PPX compared to the placebo groups, some studies did not detect any dyskinesia as a side effect of this medication. PPX was consistently associated with lower risk for developing dyskinesia compared to L-DOPA. Moreover, the presence of these symptoms in the placebo groups suggests involvement of non-PPX-related factors for developing dyskinesia. It is suggested that future research should aim at ascertaining whether cotherapy with L-DOPA, PPX dosage, and other patient characteristics are contributory factors for the development of PPX-related dyskinesia in patients with PD.

## 1. Dyskinesia in Parkinson's Disease

**Figure 1:**
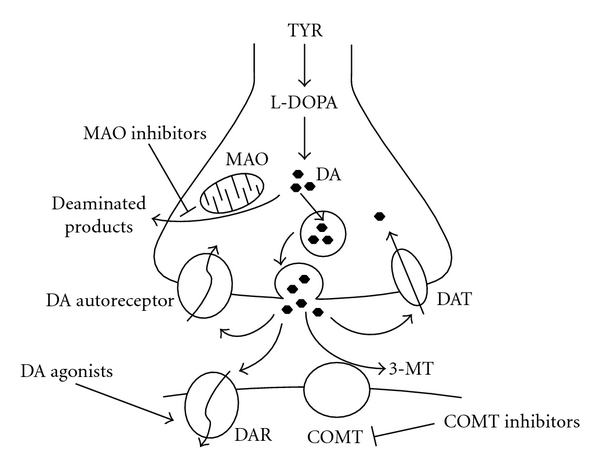
Treatment of Parkinson's disease: anti-Parkinson's medications modulate key stages of dopaminergic neurotransmission. Abbreviations: TYR: tyrosine, L-DOPA: L-3, 4-dihydroxyphenylalanine, DA: dopamine, MAO: monoamine oxidase, DAT: DA reuptake transporter, COMT: catechol-O-methyltransferase, 3-MT: 3-methoxytyramine, DAR: DA receptor. Anti-Parkinson's drugs are highlighted in bold. Pointed arrows indicate stimulatory, and closed arrows indicate inhibitory activity.

Parkinson's disease (PD) is a neurodegenerative disease characterised by motor (particularly tremor, rigidity, and bradykinesia) as well as cognitive and behavioural symptoms. The pathophysiology of PD has been related to the degeneration of nigrostriatal dopaminergic pathways [1], and this has allowed the treatment for PD to be targeted towards modulating dopamine (DA) neurotransmission (Figure 1).

Levodopa (L-DOPA) has long been the mainstay of PD treatment although over time patients on L-DOPA develop motor complications including dyskinesias, which are associated with the timing of drug administration. Dyskinesias are involuntary muscular contractions and include choreic, dystonic, myoclonic, and tic movements [2]. After 5 and 10 years of L-DOPA therapy, 91% and all of the participants in a longitudinal cohort (*N* = 99), respectively, experienced dyskinesias [3]. Another study also identified that cumulative L-DOPA dosage was significantly associated with the development of dyskinesia [4].

Given that dyskinesias has been consistently shown to negatively affect patients' quality of life [5, 6], there is considerable debate on how to forestall its onset, including initial treatment with another class of drugs: the dopamine receptor agonists (DAAs) [7, 8]. Pramipexole (PPX) belongs to this drug class and is selective for the D_2_-like receptor subfamily, particularly the D_3_ compared to the D_2_ and D_4_ subtypes [9]. Following the observation by Hauser et al. [4] that treatment with PPX was significantly associated with later onset of dyskinesia, we carried out a systematic literature review to examine the effects of PPX therapy on dyskinetic events in patients with PD.

## 2. Literature Search Methodology

This paper systematically reviews the existing evidence on the development of dyskinesia during PPX therapy for PD. We performed a literature search across the databases Medline, EMBASE and PsycInfo via the NHS Evidence tool (http://www.library.nhs.uk). We used the search terms “Parkinson*,” “dyskinesi*,” and “pramipexole”. The Cochrane Library was also searched for randomised and double-blind human trials of PPX in patients with PD. We limited our search to papers published in English language.

## 3. Pramipexole-Placebo Comparisons

The majority of the studies on PPX included in this review were comparisons with placebo only (Table 1). Six out of ten of these studies found that dyskinesia in PPX-treated patients was prevalent, at a higher rate than in the placebo group. The incidence of dyskinesia was 7.0–61.3% and 3.0–40.8% in the PPX and placebo groups, respectively [10–15]. The differences in rates of dyskinesia were between 4.9 and 20.5%. Two of these studies had follow-up data. An extension to the Lieberman et al. [10] protocol by up to 50 months was carried out, in which both PPX and placebo groups were re-titrated onto open-label PPX [16]. Out of the sample (*N* = 306), 61.1% reported dyskinesias, but there were no related discontinuations. Furthermore, UPDRS IV scores remained below the baseline values, indicating some improvements in these symptoms. Möller et al. [13] reported an open-label extension to their study of up to 57 months follow-up. Out of their cohort, 34.4% (*N* = 262) developed dyskinesias. This led to study discontinuation in 2.3% of participants. Two studies reported the incidence of dyskinesia to be higher with the placebo than the PPX groups: 0.6% versus 0.0% [17] and 6.1% versus 5.6% [18]. However, it should be noted that the strength of such evidence is weak. One study [17] was specifically designed to assess the antiparkinsonian properties in early PD, rather than the potential dyskinetic effects of pramipexole. The other one [18] found 2 dyskinetic patients in a group of 33 patients treated with placebo and 2 dyskinetic patients in a group of 36 patients treated with PPX. Of note, a further study failed to identify participants who experienced dyskinesia with PPX treatment or placebo [19, 20].

In terms of neurological scales as a measure of dyskinesia, Lieberman et al. [10] reported that % change in UPDRS IV scores was significantly (*P* < .0001) higher in the PPX group compared to the placebo group. Changes in the PDS were not statistically significant. These exact patterns in UPDRS IV and PDS scores were reproduced by Pinter et al. [11] and Möller et al. [13]. In these studies, however, the incidence of dyskinesia was higher with PPX treatment than placebo. Wermuth et al. [18] did not find significant changes in UPDRS IV or PDS scores. In a cross-over design with PPX and placebo, as well as L-DOPA infusion before and after the switch-over, Brodsky et al. [32] found that PPX treatment increased PDS scores to statistically significant levels (*P* = .05). Furthermore, L-DOPA infusion also increased peak dyskinesia scores. 

## 4. Studies with Active Comparators

### 4.1. Pramipexole Only

There have been two studies comparing different dosages and other two looking at different preparations of PPX. One of the earliest investigations on PPX was carried out by the Parkinson Study Group [21], which compared four different dosages of the drug (1.5, 3.0, 4.5, and 6.0 mg) against placebo. Another study by the same group compared different low-dose schedules of PPX [21]. Both did not detect any incidence of dyskinesia. Likewise, comparisons of immediate release (IR) and extended release (ER) PPX preparations failed to detect any incidence of dyskinesia symptoms in either treatment or placebo group [26, 27].

### 4.2. Pramipexole versus Other Dopamine Agonists

Other DAAs have been compared with PPX. Two studies reported a comparison with bromocriptine (BRC), showing that dyskinesias were found more often with DAAs compared with placebo. In terms of UPDRS IV and PDS scores, there were no significant changes in one trial [29], whereas, in the other, % UPDRS IV score changes were significantly lower in the PPX compared to the placebo group [30]. When comparing PPX and BRC, Guttman et al. [29] found that the incidence of dyskinesia between these treatment groups was approximately similar (5% difference). On the other hand, dyskinesia was found to be nearly twice as prevalent with PPX treatment compared to BRC [30].

Two studies compared pergolide (PGL) with PPX. One of these studies found that dyskinesia was equally prevalent with PPX and PGL (*N* = 1) but was not reported in the placebo group [23]. Another trial by the same group with a cross-over design found an overall lower incidence of dyskinesia with PPX treatment than PGL: 33.3% versus 37.5% [25]. Transdermally administered rotigotine (RTG) was also compared with PPX [31]. The placebo group had less dyskinesia than the active treatment groups, with PPX having slightly higher incidence than RTG (3% versus 15% versus 12%). Furthermore, this group reported that participants in the PPX group had significantly more time in the day “on” without troublesome dyskinesias compared to placebo (*P*.0429), whereas the difference between PPX and RTG was not significant.

### 4.3. Pramipexole versus L-DOPA

Only one cohort was involved with a trial comparing PPX and L-DOPA. The first report was the two-year completion of initial treatment with PPX and L-DOPA, with open-label L-DOPA for emerging disability [22]. There was a significantly lower incidence of dyskinesia in the PPX compared to the L-DOPA group: 9.9% versus 30.7%, hazard ratio (HR) 0.33 (confidence interval (CI) 0.18–0.60), *P* < .001. Some of the participants who completed the two-year trial were also enrolled for a further two-year follow-up study, with the randomised and blinded protocol maintained [24]. This showed a similar pattern of the incidence of dyskinesia, which was significantly lower in the PPX (*N* = 83) compared to the L-DOPA (*N* = 100) group: 24.5% versus 54.0%, HR 0.37 (95% CI 0.25–0.56), *P* < .001. None of the PPX cohort withdrew from follow-up due to dyskinesia, whereas dyskinesia-related discontinuation was found in 2.0% of the L-DOPA group. Some of the patients in the 2-and 4-year trials were also recruited to the open-label extension study with mean follow-up of 6.0 (SD 0.2) years, the majority of whom were in H-Y stages 2 [35]. Both the initial PPX (*N* = 108) and L-DOPA (*N* = 114) groups showed overall reductions in the incidence of dyskinesia: 20.4% and 36.8%, respectively. Despite changes in the incidence of dyskinesia in the treatment groups compared to the previous reports of this cohort, L-DOPA treatment was associated with higher events. Furthermore, disability associated with dyskinesia in the PPX group was comparably lower than that in the L-DOPA group, which was at trend-level significance (*P =*.06).

## 5. Are There Indicators for the Development of Dyskinesia with PPX Treatment?

The majority of placebo-controlled studies demonstrated that dyskinesias can develop during PPX treatment. The incidence of these events in the placebo groups ranged between 0 and 40.8%, and the differences compared to the PPX group ranged between 4.0 and 21.3%. Two studies demonstrated slightly more dyskinesia in the placebo compared to the PPX group [17, 18]. These results clearly indicate that there are non-PPX-related factors contributing to the development of dyskinesias in PD.

Treatment of L-DOPA has been proposed as an important factor for the development of dyskinesia in PD [4]. From the two trials and the long-term follow-up of their cohort [22, 24, 35], the PSG consistently demonstrated a higher incidence of dyskinesia associated with L-DOPA therapy. Furthermore, Brodsky et al. [32] showed that PPX treatment increased L-DOPA-related dyskinesias and increased the severity and duration of dyskinesia. They hypothesised that the observed effects in their study are beyond potential additive effects, given that DAAs rarely cause dyskinesia. The 2-hour infusion of therapeutic dose L-DOPA (1.0 mg/kg/hr) also produced more dyskinesia compared to subtherapeutic doses (0.5 mg/kg/hr), regardless of PPX treatment. These findings suggest that L-DOPA on its own can have some effect on dyskinesia events during PPX therapy. Although a study distinguished between L-DOPA and non-L-DOPA-treated participants, the report about adverse event-related withdrawals (including dyskinesia) in the L-DOPA group was not supplemented by information about what proportions were affected [12]. The effects of L-DOPA in other studies are also less clear because L-DOPA usage is reported for the whole sample and there is no differentiation of which patients were dyskinetic.

The literature also allows limited exploration from clinical studies whether continuous dopaminergic stimulation (CDS) is a protective factor for dyskinesia [36]. CDS is a proposed strategy to prevent fluctuations in DA transmission and therefore the development of dyskinesia [37]. Studies with PPX and other DAAs as active comparators show similar incidence of dyskinesia. In all studies with placebo comparisons, DAAs have consistently been associated with more dyskinesia events, suggesting the involvement of dopaminergic activation. In terms of the pharmacokinetic profile of PPX (Table 2), its longer half-life compared to L-DOPA makes it difficult to explain the higher incidence of dyskinesias with this treatment. In fact, the shorter half-life of L-DOPA compared to other antiparkinsonian medications (such as dopamine D2 receptor agonists) is regarded as a factor contributing to its pulsatile action and ultimately to dyskinesia [38]. Moreover, studies on the ER preparation of PPX, which produces a continuous release of active ingredient over a twenty-four-hour period [39], did not find high rates of dyskinesia. Finally, there were no differences whether the DAA was ergoline (BRC, PGL) or non-ergoline-based (PPX, RTG). It has therefore been suggested that previous exposure to L-DOPA (i.e., priming) can lead to increased susceptibility to develop dyskinesias after exposure to drugs which would not otherwise have had this effect. Specifically, pulsatile activation of type D2 dopamine receptors is reported to be the principal factor in the triggering of dyskinesias and may well be involved in the priming phenomenon [40].

Dose-ranging studies of PPX allow some degree of examination of the hypothesis that PPX-related dyskinesia may be dosage dependent. One study examining PPX at doses of 1.5, 3.0, 4.5, and 6.0 mg did not report any incidence of dyskinesia in either treatment or placebo groups [21]. Another study of different schedules of low-dose PPX (0.5 bd, 0.75 bd, and 0.5 mg td) also did not report dyskinetic events in the participants [28]. These findings suggest that dyskinesia may not be PPX dose dependent. Exploration of this hypothesis with other studies is difficult because PPX dosages are reported as a mean or range of values for the whole sample. Therefore, it is not possible to determine what doses were administered to patients with dyskinesia.

Due to insufficient details in published reports, it has also been difficult to explore whether concomitant APD usage or patient characteristics (such as illness stage, age, and duration of disease) are associated with dyskinesia. APDs were either kept constant at baseline dosages or used as add-on therapy for emerging disability and the majority of studies allowed concomitant usage. Furthermore, patient characteristics were reported as mean or range values without differentiating which patients exhibited dyskinesia. However, it is interesting to note that patients with dyskinesias were generally older (early sixties and above). The patients in PPX-only studies in which there was no incidence of dyskinesia were newly diagnosed with PD.

In terms of methodological issues, the duration of the treatment phase (i.e., titration and maintenance) has overall been adequate to allow sufficient time in detecting or establishing a timeframe for the onset of dyskinesia. The sample size has also generally been statistically viable to allow detection of clinically relevant findings, including dyskinetic adverse events, although there have been some studies with small sample sizes. Most studies also maintained some equivalency with patient characteristics in their treatment groups by carrying out block randomisations and statistical tests before and after treatment to investigate related differences in outcome measures.

More problematic issues, however, may confound conclusions about the effects of PPX on dyskinesia. In particular, some studies only reported adverse events including dyskinesia that occurred above threshold incidence, for example, ≥2%, ≥5%, or 10%. Thus, PPX-related dyskinesias may be underreported and contribute to the lower incidence in certain studies. Indeed, the studies which did not detect dyskinesia in their participants had thresholds of reporting these events at ≥5% [26–28] and 10% [19]. There have also been studies in which patients already experiencing motor fluctuations including dyskinesia were enrolled for PPX treatment. These studies examined whether PPX is a suitable adjuvant medication for L-DOPA-related dyskinesia. However, from these studies it is difficult to establish whether PPX can contribute to the development of dyskinesias since it was not described whether the incidence of dyskinesia is treatment-emergent and relevant neurological scales were not used to measure effects on these symptoms. The reporting of dyskinesia in follow-up studies also does not allow the differentiation between patients with treatment-emergent events and those who continued to experience such symptoms. Additionally, the combination of ratings of dyskinesia and neurological scales to measure effects of PPX further confound conclusions without differentiating which patients had dyskinetic events. For example, the incidence of dyskinesia may be high in the PPX group even if the scales show significant improvement associated with PPX treatment [10, 11, 13].

## 6. Conclusions

We systematically reviewed the existing evidence on the use of PPX in PD with focus on the development of dyskinesia. Treatment with PPX has overall been associated with significantly better improvement in motor and daily function compared to placebo. The majority of placebo-controlled studies demonstrated that dyskinesias can develop during PPX treatment, as these symptoms were generally (except for two studies) more prevalent compared to the placebo groups. Therefore, the evidence in support of lower incidence of dyskinesia in combination with PPX is far from convincing. However, in six studies, no dyskinesia events were reported. Four of these six studies were PPX comparisons (i.e., dose-ranging or immediate versus extended release preparation). Active comparator studies showed that the incidence of dyskinesia events was quite similar relative to other DAAs. An L-DOPA comparison study in one cohort with long-term follow-up consistently showed that PPX treatment was associated with lower risk for developing dyskinesia. These symptoms have also been reported in the placebo groups (albeit at generally lower rates), suggesting the potential involvement of non-PPX-related factors in the development of dyskinesia. It is still to be established whether L-DOPA treatment, PPX dosage, and other patient characteristics such as age or disease stage can play a role as contributory factors. Elucidation of such factors is likely to optimise the efficacy of anti-Parkinson's treatment and its compliance.

## Figures and Tables

**Table 1:** Dyskinesia with pramipexole treatment: summary of double-blind randomised-controlled trials of pramipexole in Parkinson's disease.

Disease stage*	Study	Drug regimen (N): mean daily dose, mg (SD/range)	Incidence of dyskinesia (%), change in UPDRS IV or PDS (%)	Concomitant L-DOPA ( % usage and/or mean dose, mg (SD/range))	Other concomitant APDs	Study duration	PPX group characteristics: H-Y stage, age, disease duration (mean (SD))
Early	Hubble et al. [20]	PPX (28), PL (27): 4.5	None	None	MAOBI	9 wks	1–3, 63.5 (12.3), 2.1 (2.5)
	PSG [21]	PPX (213), PL (151): fixed dose at 1.5, 3.0, 4.5, 6.0	None	PPX: 24.4%, PL: 27.5%	MAOBI	10 wks	1–4 (1.9, 0.6), 62.0 (10.9), 2.0 (1.6)
	Shannon et al. [17]	PPX (164): 3.8, PL (171): 0.375–4.5	PPX: 0%, PL: 0.6% (leading to discontinuation)	None	MAOBI	31 wks	1–3, 62.7, 1.8
	PSG [22]^†^	PPX (151): 1.5–4.5, L-DOPA (150): 300–600	PPX: 9.9%, L-DOPA: 30.7% (HR 0.33 (95% CI, 0.18–0.60); *P* < .001)-↑66.7% (PPX), ↑82.6% after O/L L-DOPA	Part of study intervention	MAOBI, amantadine, AC	23.5 mos	1–3 (82.8% ≤ 2), 61.5 (10.1), 1.5 (1.4)
	Pogarell et al. [19]	PPX (44), PL (39): 0.375–4.5	None	PPX: 11%, PL: 13%	MAOBI and amantadine	11 wks	1–3 (72.7% ≤ 2), 62.0 (10.1), 6.5 (4.0)
	Navan et al. [23]	PPX, PGL, PL (10): 4.5	PPX: 10.0%, PGL: 10.0%, PL: 0.0%	PPX: 60%, 400 (200–600), PGL: 60%, 383 (300–700), PL: 40%, 550 (300–800)	MAOBI, amantadine, and AC	3 mos	1-2 (1), 66 (55–80), 4 (0.5–10)
	Wong et al. [12]	PPX (73), PL (77): 0.375–4.5	PPX: 12.3%, PL: 5.2%	PPX: 68.5%, PL: 70.1%	None	15 wks	2.2 (0.07), 58.8 (1.28), 4.5 (0.4)
	PSG [24]^†^	PPX (83): 2.78 (1.1), L-DOPA (100): 427 (112)	PPX: 24.5.5, L-DOPA: 54.0 (HR 0.37 (95% CI 0.25–0.56); *P* < .001)	PPX: 434 (498), L-DOPA: 274 (442)	MAOBI, amantadine, AC	4 yrs	1–3 (79.5% ≤ 2), 61.1 (9.6), 1.4 (1.3)
	Navan et al. [25]	PPX (9): 3.09, PGL (8): 3.0, cross-over	PPX: 33.3%, PGL: 37.5%	52.9%; 544 (300–1000)	AC	12 wks–9 wks cross-over	1-2 (1.4), 68.4 (55–84), 3.9 (0.2–12.0)
	Barone et al. [15]	PPX (139): 2.18 (0.83), PL (148): 2.51 (1.66)	PPX: 7%, PL: 3%	PPX: 76%, PL: 74%	Amantadine, MAOBI, ACI, and ODD	12 wks	1–3 (79% ≥ 2), 67.4 (9.0), 4.0 (4.5)
	Hauser et al. [26]	PPX IR (103), PPX ER (106), PL (50): 0.375–4.5	None	PPX IR: 1.0%, PPX ER: 2.9%, PL: 14.0%	None	18 wks	1–3 (72.3% 2-3), 61.8 (8.9), 1.0 (1.3)
	Rascol et al. [27]	PPX IR (52), PPX ER (104): 1.5–4.5	None	PPX IR: 51.9%, PPX ER: 56.7%	MAOBI, COMTI, AC, and amantadine	4 wks IR + 9 wks IR/ER	1–3 (80.8% 1-2), 63.7 (9.1), 3.3 (2.0)
	PSG [28]	PPX 0.5 bd (81), 0.75 bd (73), 0.5 td (80), PL (77)	None	None	MAOBI, AC, and amantadine	12 wks	1–2.5 (89.7% 1-2), 63.3 (10.0), 2.6 (2.6)

Advanced	Guttman et al. [29]	PPX (79): 3.4, BRC (84): 22.6, PL (83)	PPX: 40%, BRC: 45%, PL: 27%; NS changes in UPDRS IV and PDS	100%	AC, amantadine, and MAOBI	9 mos	2–4, 62.9 (10.0), 0.67–36
	Lieberman et al. [10]	PPX (181), PL (179): 0.375–4.5	PPX: 61.3%, PL: 40.8%; UPDRS IV PPX > PL (*P* < .0001), PDS PPX > PL (*P = *NS)	100%; PPX: 843.4 (578.9), PL: 819.2 (466.1)	MAOBI and amantadine	31 wks	2–4 (3.0), 63.4, 9.4
	Wermuth et al. [18]	PPX (36): 4.59 (0.95), PL (33): 4.77	PPX: 5.6%, PL: 6.1%; NS change in PDS; NS change in UPDRS IV	PPX: 100% (61.1% > 600), PL: 100% (66.7% > 600)	MAOBI, ACI, and amantadine	11 wks	2–4 (91.7% 2-3),63.2 (7.9), 10.1 (5.0)
	Pinter et al. [11]	PPX (34), PL (44): 0.2–5.0	PPX: 14.7%, PL: 4.5%; UPDRS IV PPX > PL (*P =*.0092); PDS PPX < PL (*P = *NS)	PPX: 26.5% (11.8% > 600), PL: 34.1% (18.2% > 600)	MAOBI and amantadine	11 wks	2–4 (79.4% ≥ 3), 59.3 (8.3), 7.8 (4.3)
	Mizuno et al. [30]	PPX (102): 3.24 (1.33), BRC (105): 17.8 (5.8), PL (108)	PPX: 15.7%, BRC: 8.6%, PL: 5.6%; UPDRS IV PPX < PL (*P =*.006), PPX *≈* BRC (*P = *NS)	PPX: 404.9 (275.2), BRC: 377.9 (237.8), PL: 422.4 (330.3)	ACI, amantadine, ODD, and MAOBI	12 wks	2.7 (0.7), 65.5 (9.5), 4.8 (4.2)
	Möller et al. [13]	PPX (168): 3.7, PL: 0.375–4.5	PPX: 30.0%, PL: 8.7%; UPDRS IV PPX > PL (*P =*.0114), PDS (*P = * NS)	PPX: 637.7, PL: 648.8	MAOBI and AC	31 wks	1–4 (85.0% 2-3), 63.4, 7.6
	Poewe et al. [31]	PPX (200): 3.1 (1.2), RTG (201): 13.0 (3.5), PL (100)	PPX: 15%, RTG: 12%, PL: 3%; hrs “on” without troublesome dyskinesia PPX > PL (*P =*.0429), PPX < RTG (*P = * NS)	PPX: 813 (459), RTG: 795 (380), PL: 814 (398)	AC, COMTI, amantadine, and MAOBI	23 wks	2–4, 63.2 (9.7), 8.4 (4.7)
	PSG [14]	PPX (109), PL (35): 0.375–4.5	PPX: 21.1%, PL: 11.4%	100%; PPX: 278.9 (211.6), PL: 272.9 (204.1)	NR	10 wks	2–4 (2.5, 0.5), 64.8 (10.6), 6.1 (5.1)
	Brodsky et al. [32]	PPX/PL cross-over (13): 3.0	PPX ↑ PDS scores compared to baseline (*P =*.05), ↑ peak scores with L-DOPA infusion	100%, 871.2 (448.6); + infusion at 5 + 10 wks	Unclear on which APDs	10 wks–5 wks cross-over	NR, 61.9 (8.0), 10.3 (4.3)

*Studies were categorised according to the disease stage (early versus advanced, plus Hoehn and Yahr stage, where available).

Abbreviations: *N*: number of patients, SD: standard deviation, PSG: Parkinson Study Group, NR: not reported, HR: hazard ratio (risk ratio of developing dyskinesia per unit of time for patients assigned to PPX compared to risk ratio for L-DOPA), CI: confidence interval, ^†^the report by PSG [22] is an extension of the PSG [31] protocol, H-Y stage: Hoehn-Yahr stage (a staging system to describe PD progression from 0 to 5 with stages 1.5 and 2.5 in the modified version, incorporated into the UPDRS), PPX: pramipexole, PL: placebo, BRC: bromocriptine, L-DOPA: levodopa (with or without carbidopa), PGL: pergolide, RTG: rotigotine, APDs: anti-Parkinson's drugs, MAOBI: monoamine oxidase-B inhibitors (e.g., selegiline), AC: anticholinergics (e.g., orphenadrine, benzhexol), ODD: other dopaminergic drugs (e.g., droxidopa), UPDRS IV: Unified Parkinson's Disease Rating Scale part IV (complications of therapy, higher scores indicate more severe dyskinesia), which also includes subitems on dyskinesia symptoms [33], PDS: Parkinson's Dyskinesia Scale (higher scores indicate more severe dyskinesia), which rates the severity of dyskinesia according to body regions [34].

**Table 2:** Pharmacokinetic profiles of selected anti-Parkinson's drugs.

APD	Half-life (hrs)
PPX	8–12
L-DOPA/carbidopa	1–1.5
RTG	5–7
PGL	7–16
BRC	12–15

Abbreviations: APD: anti-Parkinson's drug, PPX: pramipexole, L-DOPA: levodopa, RTG: rotigotine, PGL: pergolide, BRC: bromocriptine.
